# A systematic review of health disparities research in deep brain stimulation surgery for Parkinson’s disease

**DOI:** 10.3389/fnhum.2023.1269401

**Published:** 2023-10-27

**Authors:** Adeel A. Memon, Kate Gelman, Joseph Melott, Rebecca Billings, Michelle Fullard, Corina Catiul, Svjetlana Miocinovic, Amy W. Amara

**Affiliations:** ^1^Department of Neurology, Rockefeller Neuroscience Institute, West Virginia University, Morgantown, WV, United States; ^2^School of Medicine, West Virginia University, Morgantown, WV, United States; ^3^UAB Libraries, University of Alabama at Birmingham, Birmingham, AL, United States; ^4^Department of Neurology, University of Colorado, Aurora, CO, United States; ^5^Department of Neurology, University of Alabama at Birmingham, Birmingham, AL, United States; ^6^Department of Neurology, Emory University, Atlanta, GA, United States

**Keywords:** deep brain stimulation, Parkinson’s disease, health disparities, racial disparities, gender disparities, socioeconomic disparities, age disparities

## Abstract

**Background:**

Deep brain stimulation (DBS) is the primary surgical intervention for Parkinson’s disease (PD) patients with insufficient response to medication, significantly improving motor symptoms and quality of life. Despite FDA approval for over two decades, access to this therapy remains limited. This systematic review aims to evaluate the influence of gender, race/ethnicity, socioeconomic status, and age on health disparities associated with DBS for PD, providing an overview of current research in this field.

**Methods:**

A systematic literature search was conducted in PubMed/MEDLINE, Embase, Web of Science and Cochrane databases from 1960 to September 12th, 2023, following Preferred Reporting Items for Systematic Reviews and Meta-Analysis guidelines. Studies that examine the disparities in accessing DBS among patients with PD were included, comparing different demographic factors. Findings were synthesized and presented narratively to identify and understand DBS disparities.

**Results:**

After screening for relevance, 25 studies published between 1960 and 2023 were included, with 16 studies meeting full-text review criteria. While reviewing the references of the 16 articles, two additional studies were included, bringing the total number of included studies to 18. Most studies originated from the United States (44%). The identified studies were categorized as identifying disparities, understanding disparities, or reducing disparities. The majority focused on identifying disparities (72%), while fewer studies delved into understanding the underlying factors (28%). No studies evaluated strategies for reducing disparities. The findings indicate that elderly, female, and Black people, as well as those from low socioeconomic backgrounds and developing countries face greater obstacles in accessing DBS for PD.

**Conclusion:**

This study highlights factors contributing to disparities in DBS utilization for PD, including race, gender, and socioeconomic status. Public health policymakers, practitioners, and clinicians should recognize these inequalities and work toward reducing disparities, particularly among vulnerable populations.

## Introduction

Reducing health disparities is a critical goal in healthcare, aiming to achieve equitable access and outcomes for all individuals, regardless of their demographic or socioeconomic background. In the context of deep brain stimulation (DBS) for Parkinson’s disease (PD), addressing health disparities becomes particularly important due to the potential impact on patients’ quality of life and disease management. Numerous randomized clinical trials (Perestelo-Pérez et al., 2014) have established the superiority of DBS over medication management in patients with PD. Moreover, considerable research has been dedicated to investigating DBS’s mechanisms and advancements, particularly in white men (Lozano et al., 2019). However, there is a limited focus on expanding the accessibility of DBS to a broader patient population. Understanding how to make DBS more accessible is of utmost importance for future healthcare service planning, especially considering the projected rise in PD incidence within the next two decades to over 17 million globally (Dorsey et al., 2018).

Health disparities research has witnessed significant growth across various medical disciplines, consistently revealing associations between factors such as minority race, low socioeconomic status, and rural place of residence with poorer health outcomes (National Academies of Sciences, Engineering, and Medicine, Health and Medicine Division, Board on Population Health and Public Health Practice, Committee on Community-Based Solutions to Promote Health Equity in the United States, 2017). National initiatives are underway to address the high rates of preventable diseases among ethnic minorities, which are projected to incur an estimated cost of $50 billion annually to the healthcare system by 2050 (Waidmann, 2009). Implementing the Affordable Care Act (ACA) is a notable example, as it expanded coverage to over 20 million previously uninsured individuals and facilitated access to preventive services (Center on budget and policy priorities, 2019). However, while insurance expansion has demonstrated its ability to improve access to care, it only comprehensively addresses some of the patient-, provider-, and system-level factors contributing to health disparities. Therefore, further efforts are required to identify and address the underlying causes of these disparities beyond the scope of insurance expansion.

This study aims to systematically review the available evidence on DBS-related health disparities for PD populations. The primary focus is to review the research to date and describe findings on essential determinants of health inequity. These determinants include race, gender, socioeconomic status, and age, which have been examined within the literature on DBS. Additionally, we have also described potential solutions to address these disparities. Our study postulated a need for more research examining healthcare disparities across various domains in the availability of DBS for PD. Furthermore, we hypothesized that existing literature would focus on identifying disparities rather than developing strategies to mitigate and alleviate them.

## Methods

### Search strategy and study selection

A systematic review search was conducted by a medical librarian on September 12, 2023, in the following databases: Embase (via Elsevier), PubMed, Cochrane Library, and Web of Science. The searches followed the Preferred Reporting Items for Systematic Reviews and Meta-Analyses (PRISMA) checklist (Moher et al., 2009) and focused on disparities in access to DBS surgery for patients with PD.

A combination of database-specific subject headings and keywords were used in the search strategies. The concepts covered included (1) health disparities OR vulnerable populations: (“health disparity” [subject term] OR “vulnerable population” [subject term] OR “social determinants of health” [subject term] OR “disparit*” [keywords] OR “discriminat*” [keywords] OR “underrepresent*” [keywords] OR “underserved” [keywords] OR “marginalized” [keywords] OR inclusiv* [keywords]) AND (2) deep brain stimulation surgery: (“deep brain stimulator” [subject term] OR “brain depth stimulation” [subject term] OR “deep brain stimulation electrode” [subject term] OR “DBS” [keywords] OR “deep-brain” [keywords] OR “brain-depth” [keywords] NEAR/3 “surger*” [keywords] OR “stimulat*” [keywords] OR “procedur*” [keywords]) AND (3) Parkinson Disease: (“Parkinson Disease” [subject term] OR “PD” [keywords] OR “Parkinson*” [keywords] OR “hemiparkinsonism” [keywords]). No date limits were applied. Specific study types were incorporated into the search strategies for PubMed, Embase, Cochrane Library, and Web of Science. Exact search strategies used for each database are included in the Supplementary Table S1.

Inclusion criteria required that articles were (1) peer-reviewed research studies, (2) included individuals diagnosed with PD, (3) studies specifically focused on DBS as a therapeutic intervention for PD, (4) incorporation of studies that investigate the availability, utilization, or accessibility of DBS; (5) studies that present findings on disparities, inequalities, or variations in the availability or accessibility of DBS for PD; (6) consideration of disparities arising from demographic factors, socioeconomic status, race/ethnicity, gender, age or other pertinent variables; (7) studies published in the English language.

Exclusion criteria included the following: (1) wrong patient population; (2) studies with wrong outcomes that do not provide data on racial, gender, socioeconomic and age disparities in DBS availability for PD; (3) studies with poor methodological quality or insufficient data to assess disparities or commentaries only. These exclusion criteria helped to focus articles selected on access disparities in DBS in patients with PD.

### Risk of bias assessment

Using Joanna Briggs Institute’s (JBI) (Moola et al., 2020) standardized critical appraisal instruments for observational studies, the quality of eligible studies was independently assessed by two investigators (KG and JM). This rigorous assessment aimed to ensure the internal validity of the review’s findings and mitigate the potential influence of confounded or biased statistics. The selected studies were categorized into following study types: cross-sectional studies, retrospective cohort studies, case–control studies, and reviews. Specific checklists corresponding to each study type were utilized for assessment purposes. The evaluation checklists consisted of 8, 11, 10 and 11 questions for cross-sectional, retrospective cohort, case–control studies, and reviews, respectively (Moola et al., 2020). Responses to the checklist items were categorized as “Yes”, “No,” “Unclear,” or “Not applicable.” A score of “1” was assigned to “Yes” responses, while “0” was assigned to “No,” “cannot be answered,” or “not applicable” responses. The quality score for each study was calculated as a percentage and reported accordingly. The final score for each study was determined through consensus between the two evaluators (Table 1). In the event of any disagreements between the investigators, resolution was sought by engaging in discussions with another investigator (AAM).

**Table 1:** Risk of bias assessment.

Bias assessment of**Cross-sectional studies** using JBI critical appraisal checklist										
Study	Q1	Q2	Q3	Q4	Q5	Q6	Q7	Q8	Score	Score %
Chan et al. (2014)	Y	Y	Y	Y	Y	Y	Y	Y	8	100
Cramer et al. (2022)	Y	Y	Y	Y	Y	Y	Y	Y	8	100
Dorritie et al. (2023)	Y	Y	Y	Y	Y	Y	Y	Y	8	100
Hamid et al. (2021)	Y	N	Y	N	U	N	NA	Y	3	37.5
Henriksen et al. (2020)	Y	Y	Y	Y	Y	Y	Y	Y	8	100
Jost et al. (2022)	Y	Y	Y	Y	Y	U	U	Y	6	75
Jourdain and Schechtmann (2014)	Y	Y	Y	Y	Y	NA	NA	Y	6	75
Meng et al. (2023)	Y	Y	Y	Y	Y	Y	Y	Y	8	100
Setiawan et al. (2006)	Y	Y	Y	Y	N	N/A	Y	Y	6	75
Shpiner et al. (2019)	Y	Y	Y	Y	N	N/A	Y	Y	6	75
Watanabe et al. (2022)	Y	Y	Y	Y	U	U	Y	Y	6	75
Average score										83%
Y, Yes; N, No; U, Unclear; N/A, Not Applicable. Q1: Were the criteria for inclusion in the sample clearly defined? Q2: Were the study subjects and the setting described in detail? Q3: Was the exposure measured in a valid and reliable way? Q4: Were objective, standard criteria used for measurement of the condition? Q5: Were confounding factors identified? Q6: Were strategies to deal with confounding factors stated? Q7: Were the outcomes measured in a valid and reliable way? Q8: Was appropriate statistical analysis used?										

Bias assessment of**Retrospective cohort studies** using JBI critical appraisal checklist													
Study	Q1	Q2	Q3	Q4	Q5	Q6	Q7	Q8	Q9	Q10	Q11	Score	Score %
Chandran et al. (2014)	Y	Y	Y	N	N/A	Y	Y	Y	Y	N/A	Y	8	72.7
Deshpande et al. (2022)	Y	Y	Y	N	U	Y	Y	Y	U	U	Y	7	63.6
Skelton et al. (2023)	Y	Y	Y	Y	Y	Y	Y	Y	Y	N/A	Y	10	90.9
Willis et al. (2014)	Y	Y	Y	Y	Y	Y	Y	N/A	N/A	N/A	Y	8	72.7
Average score													75%
Y, Yes; N, No; U, Unclear; N/A, Not Applicable. Q1: Were the two groups similar and recruited from the same population? Q2: Were the exposures measured similarly to assign people to both exposed and unexposed groups? Q3: Was the exposure measured in a valid and reliable way? Q4: Were confounding factors identified? Q5: Were strategies to deal with confounding factors stated? Q6: Were the groups/participants free of the outcome at the start of the study (or at the moment of exposure)? Q7: Were the outcomes measured in a valid and reliable way? Q8: Was the follow up time reported and sufficient to be long enough for outcomes to occur? Q9: Was follow up complete, and if not, were the reasons to loss to follow up described and explored? Q10: Were strategies to address incomplete follow up utilized? Q11: Was appropriate statistical analysis used?													

Bias assessment of**Case control studies** using JBI critical appraisal checklist												
Study	Q1	Q2	Q3	Q4	Q5	Q6	Q7	Q8	Q9	Q10	Score	Score %
Crispo et al. (2020)	Y	Y	Y	Y	Y	Y	Y	Y	Y	Y	10	100
Average score												100%
Y, Yes; N, No; U, Unclear; N/A, Not Applicable. Q1: Were the groups comparable other than the presence of disease in cases or the absence of disease in controls? Q2: Were cases and controls matched appropriately? Q3: Were the same criteria used for identification of cases and controls? Q4: Was exposure measured in a standard, valid and reliable way? Q5: Was exposure measured in the same way for cases and controls? Q6: Were confounding factors identified? Q7: Were strategies to deal with confounding factors stated? Q8: Were outcomes assessed in a standard, valid and reliable way for cases and controls? Q9: Was the exposure period of interest long enough to be meaningful? Q10: Was appropriate statistical analysis used?												

Bias assessment of**Systematic Reviews** using JBI critical appraisal checklist													
Study	Q1	Q2	Q3	Q4	Q5	Q6	Q7	Q8	Q9	Q10	Q11	Score	Score %
Hariz et al. (2011)	Y	Y	Y	N	Y	U	U	Y	N	Y	Y	7	63.6
Jamora and Miyasaki (2017)	Y	Y	Y	Y	U	U	U	NA	N	Y	Y	6	54.5
Average score													59.1%
Y, Yes; N, No; U, Unclear; N/A, Not Applicable. Q1: Is the review question clearly and explicitly stated? Q2: Were the inclusion criteria appropriate for the review question? Q3: Was the search strategy appropriate? Q4: Were the sources and resources used to search for studies adequate? Q5: Were the criteria for appraising studies appropriate? Q6: Was critical appraisal conducted by two or more reviewers independently? Q7: Were there methods to minimize errors in data extraction? Q8: Were the methods used to combine studies appropriate? Q9: Was the likelihood of publication bias assessed? Q10: Were recommendations for policy and/or practice supported by the reported data? Q11: Were the specific directives for new research appropriate?													

### Data extraction

Given the considerable diversity observed in study settings, participant characteristics, methodologies, exposure variables, and outcome measures across the included studies, a Meta-analysis was deemed inappropriate. Instead, a synthesis of findings was conducted by systematically extracting and organizing information from each manuscript. The results from the individual studies were presented and discussed using a narrative approach, complemented by the presentation of key findings in tabular formats. The conducted studies were systematically grouped into three primary categories, with the first category centered on the recognition and examination of disparities, the second delving into the exploration of the factors that underpin these disparities, and the third dedicated to the formulation and assessment of strategies aimed at mitigating these inequalities. This approach allowed for a comprehensive overview and interpretation of the collective evidence without relying on quantitative pooling methods.

## Results

**Figure 1:**
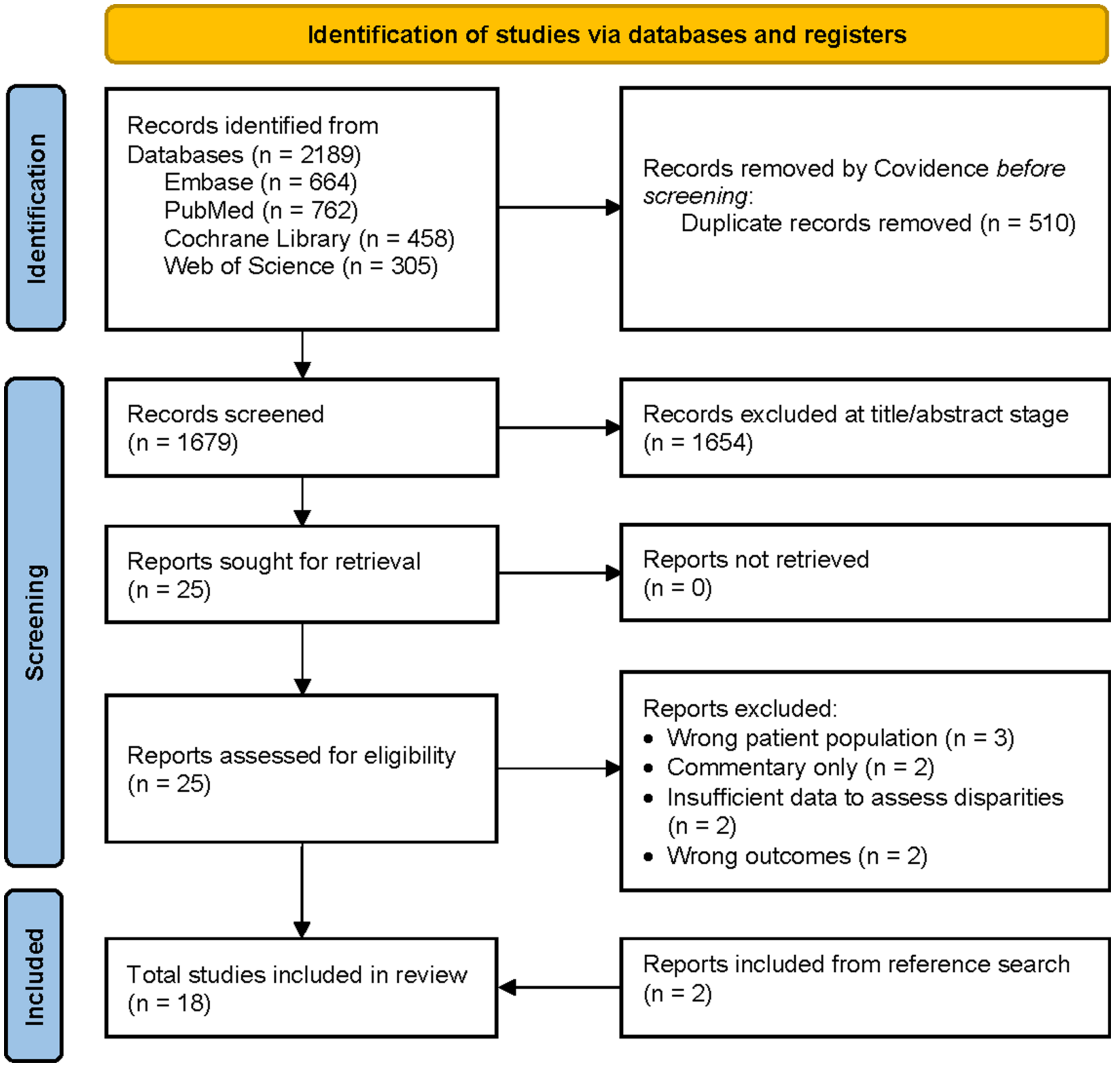
PRISMA flow diagram.

The process for manuscript selection is displayed in Figure 1. A total of 2,189 articles were identified in the literature review. Covidence removed 510 duplicate citations, leaving 1,679 unique references to be screened at the title/abstract stage. Two reviewers (KG and JM) independently conducted the initial review of the deduplicated titles and abstracts from the literature review for relevance, which led to the elimination of 1,654 citations (Figure 1). Subsequently, articles that met the initial inclusion criteria underwent a second review stage to identify the relevant 25 articles and evaluate study design quality. This process led to the exclusion of another 9 references, resulting in a set of 16 articles judged to be highly relevant and meeting the inclusion/exclusion criteria. While reviewing the references of the 16 articles, two additional studies were included, bringing the total number of included studies to 18. In cases where disagreements arose between the reviewers, a third reviewer (AAM) conducted an independent review to resolve them. The detailed process of study selection, including the reasons for excluding articles after the full-text review, is outlined in Figure 1.

The assessment of study quality in the included articles revealed that cross-sectional studies (*N* = 11) had an average quality score of 83%, retrospective cohort studies (*N* = 4) scored 75%, reviews (*N* = 2) scored 59.1% and case–control studies (*N* = 1) achieved a perfect score of 100%.

**Figure 2:**
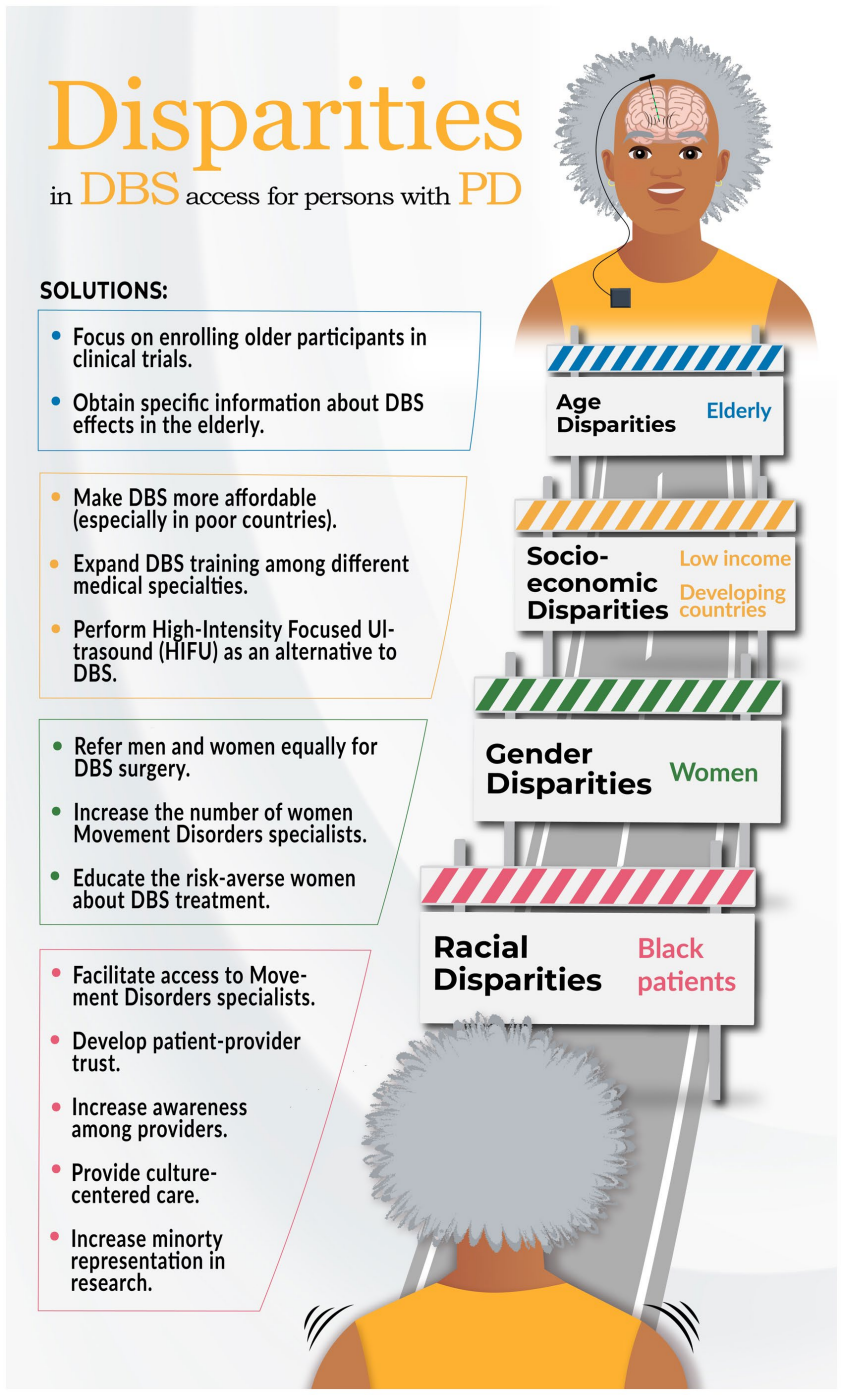
Infographic on health disparities research in DBS surgery for PD and potential solutions.

Regarding the categorization of the research, our analysis revealed that 13 studies (72%) were classified in the disparity detection category, focusing on identifying patterns and associations. In contrast, 5 studies (28%) were classified as the understanding disparities category, aiming to comprehend the underlying causes. However, no studies were identified in the reducing category, which entails developing interventions or strategies to mitigate the identified issues. Figure 2 in the infographic offers a concise overview of the paper’s content.

### Racial disparity

Our review revealed that the availability of DBS among Black individuals is lower (Chan et al., 2014; Willis et al., 2014; Cramer et al., 2022; Dorritie et al., 2023; Skelton et al., 2023). Table 2 provides details of the included studies. The following section presents a summary of studies categorized within the understanding phase of research, shedding light on factors contributing to the observed disparities.

**Table 2:** Summary of studies on health disparities research in DBS surgery for PD.

Study	Type of study	Study purpose/aim	Study sample	Exposure primary (secondary)	Testing phase	Main results % findings
Chan et al. (2014)	∙ Cross-sectional National Inpatient Sample ∙ USA	To examine deep brain stimulation use in Parkinson disease to determine demographic, clinical, and socioeconomic variables that influence DBS use.	*N* = 2,408,302 African American *N* = 114,168 Other *N* = 2,294,134	Race (insurance status)	Detecting	African Americans accounted for only 4.7% of all PD discharges and 0.1% of DBS-related discharges.
Chandran et al. (2014)	∙ Cohort ∙ Single-center ∙ India	To investigate differences in gender and clinical characteristics in patients referred for DBS, and the outcomes when resources are limited.	*N* = 51 Female *N* = 19 Male *N* = 32	Gender	Detecting	Despite no gender difference in age of onset, duration, or severity of motor symptoms prior to DBS placement, women were on lower doses of dopaminergic medications. Women also had worse depression and emotional scores.
Cramer et al. (2022)	∙ Cross-sectional ∙ National Inpatient Sample ∙ USA	To determine whether racial and socioeconomic disparities in the use DBS for PD have improved.	*N* = 4,662,026 Black *N* = 255,284 Other *N* = 4,406,742	Race	Detecting	Despite an overall increase in the odds of DBS placement from 2002 to 2018, Black patients remained five times less likely to undergo DBS than White patients.
Crispo et al. (2020)	∙ Case control ∙ Institute for Clinical Evaluative Services (ICES) datasets ∙ Canada	To examine sociodemographic characteristics and health care utilization impacts on DBS surgery for PD.	*N* = 2,207 Northern Ontario *N* = 133 Southern Ontario *N* = 2059	Sociodemographic region	Detecting	Before controlling for medication use, patients in northern Ontario were more likely to receive DBS than patients in southern Ontario. Patients living in neighborhoods with more visible minorities had less access to DBS compared to patients in predominantly white neighborhoods.
Deshpande et al. (2022)	∙ Cohort ∙ Single-center ∙ USA	To assess whether a gender disparity exists from diagnosis to DBS surgery.	*N* = 53 Female *N* = 18 Male *N* = 35	Gender	Understanding	There was no gender difference in the timeline from diagnosis to DBS surgery.
Dorritie et al. (2023)	∙ Cross-sectional ∙ National Inpatient Sample ∙ USA	To identify racial and ethnic disparities in DBS utilization in those hospitalized for ET, PD, and dystonia.	*N* = 21,963 Black *N* = 1,469 Other *N* = 20,494	Race (Insurance Status, Socioeconomic Status, Sociodemographic Region)	Detecting	Between 2012–2018, Black patients with PD were 7 times less likely to receive DBS compared to White patients.
Hamid et al. (2021)	∙ Continent-wide Survey distributed to physicians specializing in PD across Africa	To investigate availability, affordability, and insurance coverage of PD therapies and services across Africa.	*N* = 28	Sociodemographic Region	Understanding	DBS was available in 5 of the 28 African countries represented.
Hariz et al. (2011)	∙ Systematic literature review	To evaluate the gender distribution of patients with PD who receive STN DBS.	*N* = 3,880 Female *N* = 1,435 Male *N* = 2,445	Gender (region)	Detecting	The proportion of male patients receiving DBS appears to exceed the reported male-to-female ratio among patients with PD.
Henriksen et al. (2020)	∙ Cross-sectional study ∙ National Patient Register ∙ Denmark	To investigate access to device-aided therapy for PD across Denmark.	*N* = 612	Gender	Detecting	Access to device-aided therapy for PD is unequally distributed across regions in Denmark.
Jamora and Miyasaki (2017)	∙ Pragmatic review ∙ Philippines	To identify treatment gaps in the care of PD patients.	N/A	Socioeconomic status (region)	Detecting	There are only 3 neurosurgeons in the Philippines. DBS is only available in two medical centers in Manila. Lack of neurologist care and need for self-funding limit the access of DBS treatment.
Jost et al. (2022)	∙ Cross-sectional ∙ Single-center ∙ Germany/UK	To examine gender proportions from referral to DBS surgery, and differences in baseline and post-operative outcomes.	*N* = 316 Male *N* = 214 Female *N* = 102	Gender	Detecting	Although women had longer duration of disease and greater dyskinesia, they were underrepresented in DBS referrals.
Jourdain and Schechtmann (2014)	∙ Survey ∙ Worldwide	To investigate the worldwide practice of DBS for PD.	*N* = 353	Socioeconomic Status	Detecting	Public health systems provide opportunity for surgical procedures in high-income countries, while lower income countries require financing by the individual resulting in more often ablative procedures.
Meng et al. (2023)	∙ Cross-sectional ∙ Multi-center ∙ China	To investigate utilization, surgical populations, centers, coverages, regional balance, and influential factors of DBS in PD.	*N* = 38,122	Sociodemographic Region	Understanding	More patients receive DBS surgery in provinces in the eastern and central regions of China with higher socioeconomic status and greater insurance availability.
Setiawan et al. (2006)	∙ Cross-sectional ∙ Single-center ∙ Canada	To determine whether gender proportions were respresented in DBS referrals, and identify reasons patients did not recieve DBS.	*N* = 91 Male *N* = 63 Female *N* = 28	Gender	Detecting	With equal amounts of male and female patients with movement disorders, just 31% of those referred for surgery were women.
Shpiner et al. (2019)	∙ Cross-sectional ∙ Single-center ∙ USA	To determine if a gender disparity exist in DBS surgery for PD, and the reasons at a single health system.	*N* = 3,251 Male *N* = 2013 Female *N* = 1,237	Gender	Understanding	Women were more likely to refuse DBS surgery due to personal decision.
Skelton et al. (2023)	∙ Cohort ∙ Single-center ∙ USA	To identify sources of racial disparity in DBS for PD.	*N* = 209 Black *N* = 10 Other *N* = 199	Race (Insurance Status)	Detecting	Disparity in access to DBS among Black PD patients occurs in the clinical process prior to evaluation for DBS candidacy (at or before the referral stage).
Watanabe et al. (2022)	∙ Cross-sectional ∙ Single-center ∙ USA	To characterize the PD population and disparities of DBS use in AA subgroups and NHPI patients.	*N* = 4,215 NHPI *N* = 409 Other *N* = 3,806	Race (Gender)	Understanding	There is an overrepresentation of males receiving DBS, most notably in the native Hawaiian or pacific islander cohort which 100% were male.
Willis et al. (2014)	∙ Cohort ∙ Medicare research-identifiable files ∙ USA	To identify sociodemographic, clinical, and physician/practice factors in DBS.	*N* = 665,765	Sociodemographic	Detecting	Within Medicare beneficiaries, Black patients, women, and those outside of the top quartile SES neighborhoods were less likely to receive DBS.

One pioneering retrospective study investigated potential barriers to DBS access within the Black population (Chan et al., 2014). Analyzing a National inpatient sample (NIS) in the United States, 240,8302 PD discharges from 2002 to 2009 were examined, among which 18,312 discharges were associated with DBS. Notably, Black patients accounted for 4.7% of all PD discharges, but only 0.1% of DBS-related discharges. Utilizing the Hierarchical Logistic Regression Model, the authors identified Medicaid utilization as a predictor of reduced DBS utilization in the Black population compared to those with private insurance or Medicare coverage. Similar results were found in a recent study that also queried the NIS database for United States hospitalizations from 2012 to 2018. It revealed that Black patients were less likely to receive DBS, primarily due to insurance and low income (Dorritie et al., 2023).

Other investigations aimed to assess whether racial disparities in DBS utilization for PD have improved over time (Cramer et al., 2022). One study utilized the NIS data spanning from 2002 to 2018. Despite observing an overall increase in the odds of DBS placement during the study period, the researchers discovered that Black patients were still five times less likely to undergo DBS than White patients.

Another study sought to investigate potential factors contributing to the racial treatment gap in DBS access during the preoperative surgical workup, with a specific focus on the experience of a single institution (Skelton et al., 2023). Through a retrospective analysis, this study examined all patients diagnosed with PD who underwent evaluation for DBS at Emory between 2016 and 2020. Despite the racial diversity observed in the metropolitan area served by the institution, DBS was underutilized in Black patients with PD. Importantly, this treatment gap was not found to be attributable to factors within the preoperative surgical selection process. The authors speculated that the discrepancy in DBS accessibility among PD patients of Black race arises during the clinical process preceding the evaluation for DBS candidacy.

### Gender disparity

Our current review found a gender disparity (Hariz and Hariz, 2000; Setiawan et al., 2006; Willis et al., 2014; Shpiner et al., 2019; Cramer et al., 2022; Deshpande et al., 2022; Jost et al., 2022; Watanabe et al., 2022) and suggested that the proportion of male patients undergoing STN DBS appears to exceed the reported 1.48: 1 male-to-female ratio among patients with PD (Katz et al., 2011; Moisan et al., 2016; Shpiner et al., 2019). Three research studies sought to elucidate the factors underlying the observed gender disparity.

One retrospective study (Shpiner et al., 2019) analyzed data from a single center, investigating a cohort of 3,251 patients diagnosed with PD. Among this cohort, 207 individuals were referred for DBS surgery, with 100 ultimately undergoing the procedure. Of the 107 who did not have DBS, women were less inclined to undergo DBS surgery, primarily due to their personal preference, while men were more prone to being lost to follow-up.

Deshpande and colleagues conducted a single-center, retrospective cohort study to examine the potential gender disparities in the time interval between the initial diagnosis of PD and the utilization of DBS therapy (Deshpande et al., 2022). The researchers analyzed gender differences in the median duration between the date of diagnosis, consultation for DBS, and the actual DBS surgery dates. The results of the study revealed no statistically significant differences between men and women in the interval from the diagnosis to DBS surgery for PD cases.

Gender disparities in referrals for DBS surgery among individuals with PD were comprehensively studied in a cross-sectional and longitudinal, prospective, observational, controlled, quasi-experimental, and international multicenter components (Jost et al., 2022). The findings revealed a significant underrepresentation of women with PD in the referral process compared to the general PD population, with a gender ratio of men to women of 2.1:1, higher than the ratio observed in PD diagnosis. The study identified various reasons for women not undergoing DBS surgery, despite positive indications during evaluations. These reasons included patients wishing for an additional period of reflection, patient preferences for further medical optimization, newly diagnosed or worsened preexisting comorbid diseases, language barriers, and undisclosed personal reasons. Additionally, general practitioners and neurologists referred fewer women than men for DBS evaluations, indicating a gender bias in referral patterns. The authors speculated that hospital medical staff might contribute to the observed gender disparities due to implicit or explicit bias since all surgical candidacy assessments were conducted in an inpatient care setting. As inpatient care setting allowed ample time to convey the rationale and clinical reasoning for DBS treatment when positive indication evaluations were present.

### Socioeconomic and geographic disparities

Geographic factors, including limited availability of specialized healthcare facilities in specific regions, can influence disparities in the utilization of DBS. For instance, a review by Jamora and Miyasaki identified a scarcity of movement disorders specialists in the Philippines, with only 9 specialists serving a population of 100.98 million (Jamora and Miyasaki, 2017). Moreover, DBS services were solely accessible in the city of Manila. Similarly, in African countries, which are projected to surpass the combined population of North America, Europe, Latin America and the Caribbean, and Oceania by year 2050 (World Health Organization, 2017), DBS was only available in Egypt, Morocco, and South Africa, with occasional availability in Algeria and Tunisia (Hamid et al., 2021). However, the high cost of DBS due to lack of insurance rendered it unaffordable for most patients in these regions. Furthermore, a large-scale multicenter study utilized data from a national census spanning 74 Chinese centers (Meng et al., 2023) and similarly found that the eastern regions had significantly larger PD populations undergoing DBS compared to provinces in the western region. This discrepancy was attributed to the fact that provinces located in the eastern region demonstrated notably higher gross domestic products when compared to their counterparts in the western and northwestern regions (Meng et al., 2023).

In a survey conducted by Jourdian and Schechtmann involving neurosurgeons from 51 countries who had performed surgical procedures on 13,200 patients in 2009, it was observed that public healthcare systems often financed surgical procedures for PD making DBS more accessible (Jourdain and Schechtmann, 2014). Conversely, in both lower and upper-middle-income countries, patients frequently self-financed their surgeries and primarily opted for ablative surgeries rather than DBS.

Surprisingly, despite free public healthcare systems, health disparities in accessing DBS for PD persist. A study conducted in Denmark revealed the existence of barriers that result in unequal access to DBS based on factors such as age, gender, marital status, and socioeconomic status (Henriksen et al., 2020). Their findings indicated that PD patients who were male, below 70 years of age, had a partner, and possessed higher levels of education (indicative of higher socioeconomic status) were more likely to receive DBS than others. Similarly, a study conducted in Ontario, Canada examined 46,237 PD patients, among whom 543 underwent DBS surgery (Crispo et al., 2020). The Canadian study identified regional disparities, as patients residing in northern Ontario were more likely to receive DBS surgery than those in southern regions. Additionally, patients residing in neighborhoods with a higher concentration of visible minorities were less likely to receive DBS surgery than those in predominantly white neighborhoods. Furthermore, regular neurologist care and multiple PD medications were positively associated with the likelihood of DBS surgery, emphasizing the importance of access to specialists in reducing disparities related to access to DBS.

### Age disparity

PD primarily affects the elderly, yet clinical trials investigating DBS often inadequately represent this demographic, and the use of DBS in elderly patients remains understudied (Krack et al., 2003; Deuschl et al., 2006). After reviewing the available literature, we found no studies addressing age disparities, which emphasizes the need for more research in this field. Additionally, Delong et al.’s study showed that older patients with PD (> 75 years) selected for DBS surgery experienced similar 90-day complication risks as younger patients, suggesting that age alone should not be the sole factor for excluding candidates from DBS treatment (DeLong et al., 2014).

## Discussion

Health disparities in DBS for PD need significantly more attention. While the benefits of DBS for PD have been extensively documented, there is still a lack of comprehensive understanding regarding the extent and nature of health disparities in DBS utilization across diverse populations. In contrast to our initial hypothesis, our analysis revealed that the majority of studies focused on identification of disparities, with only a limited number addressing understanding the factors underlying the disparities. These studies demonstrated that women, Black patients, individuals from low socioeconomic status backgrounds, and those residing in developing countries were particularly vulnerable to disparities in DBS access. The disparities remained unexplored among older patients despite PD primarily affecting the older population. As a result, our discussion will primarily center around the racial, gender and socioeconomic factors contributing to these disparities, shedding light on the complex interplay of various elements of inequity within the context of DBS utilization for PD.

### Racial disparities

The findings in this comprehensive review confirm that DBS is often underutilized among Black patients and other racial minorities with PD. Moreover, the racial disparity in DBS utilization has remained relatively unchanged over the past decade (Cramer et al., 2022), with most data focusing on Black and White populations collected exclusively in the US, and there is no available data on other races. While most studies conducted thus far have focused on identifying the existence of this disparity, only a few have delved into the underlying causes and factors contributing to this phenomenon.

The findings of Skelton and colleagues showing a significant underutilization of DBS among Black patients with PD at an early stage, with a limited number of Black patients being referred for evaluation (Skelton et al., 2023) suggest the presence of potential unconscious or implicit bias, as well as other systemic factors contributing to the observed disparity (Wilson and Din, 2018). Consequently, there is a clear need for interventions to modify physician behavior and improve the referral and selection processes. Moreover, it is essential to consider the role of marketing in exacerbating the upstream disparities, as Black patients are less likely to be exposed to direct-to-consumer marketing efforts than their white counterparts (Lee and Begley, 2010). Addressing the disparity in the referral system requires implementing various strategies, such as continuing education, computerized decision support systems, and reminders, as these strategies have shown effectiveness in modifying behavior (Mostofian et al., 2015). Incorporating these strategies into medical training and enhancing access to movement disorders specialists can alleviate the disparities in DBS utilization (Devine et al., 2012; Maina et al., 2018).

Disparities in surgical outcomes among racial groups can potentially contribute to the observed racial disparity using DBS. One notable factor contributing to these disparities is the long-standing mistrust and deep-rooted distrust that Black patients harbor toward the healthcare system, which can be traced back to historical events such as the well-known Tuskegee syphilis study (CDC, 1932). This pervasive mistrust and fear significantly impact Black patients’ attitudes and behaviors, leading to greater hesitancy and reluctance to undergo DBS surgery (Scharff et al., 2010). This distrust underscores the critical importance of acknowledging and thoroughly studying the various factors that hinder access to care for racial minorities. However, several studies have reported no significant differences in surgical complications related to DBS for PD between Black and White patients (Fana et al., 2019; Skelton et al., 2023). Educating healthcare providers and raising awareness among Black patients about these comparable surgical outcomes is crucial for fostering equity in DBS utilization. Ensuring that both patients and healthcare professionals are well-informed can address some of the barriers that contribute to the observed racial disparities in DBS treatment.

Socioeconomic factors may significantly contribute to the observed disparities in DBS utilization among racial minorities. Research conducted by Chan and colleagues revealed that Black patients increased reliance on Medicaid predisposed them to the DBS disparity (Chan et al., 2014). Surprisingly, white patients utilizing Medicaid received significantly more DBS surgeries than Black patients using private insurance and Medicare, indicating that a distinct combination of Medicaid and race/ethnicity is responsible for the observed access disparity (Chan et al., 2014). Further investigations are warranted to comprehensively understand the relationship between socioeconomic factors, cultural factors, and Medicaid utilization in the context of DBS access (Eskandar et al., 2003; Katz et al., 2011; Dorritie et al., 2023). Exploring the complex interplay between these factors can provide valuable insights into the mechanisms underlying the DBS disparity among racial minorities and inform the development of targeted strategies to mitigate these disparities.

It is important to note that comorbidities do not appear to be the underlying factor contributing to the racial disparity in DBS utilization (Cramer et al., 2022). Black patients with PD tend to under-report motor symptoms and receive diagnoses later in their illness, often perceiving PD as a natural part of aging and thus being less inclined to seek treatment (Dahodwala et al., 2011). Furthermore, the underrepresentation of diverse ethnicities in the PD diagnosis criteria, primarily derived from analyses of Caucasian populations, may contribute to the observed disparity (Schneider et al., 2009). One potential approach to addressing this disparity is to increase the representation of racial minorities in the medical field, particularly in specialties such as movement disorders and neurosurgery. Patient-physician racial/ethnic concordance enhances communication and patient satisfaction (Saha et al., 1999), potentially facilitating Black patients’ willingness to disclose their medical conditions to healthcare providers. Implementation of culture-centered care, providing trained language interpreters, incorporating faith-based resources, and offering incentives to manufacturers and medical providers that align with the racial distribution of the general population are some potential strategies to promote equity in healthcare for racial minorities (Ojukwu et al., 2020). Further research is necessary to investigate the role of comorbidities and surgical outcomes in contributing to the racial disparity in DBS utilization. Addressing these factors and providing culturally sensitive care are crucial steps toward reducing disparities in access to DBS for racial minority populations.

### Gender disparities

This comprehensive review of the literature highlights disparities based on gender in the utilization of DBS for PD, which aligns with prior research examining disparities in access to invasive treatments for cardiac and gastrointestinal conditions (Hvelplund et al., 2010; Chibber and Baranchuk, 2020). Several studies have identified a notable gender disparity in the utilization of DBS for PD, with a significantly higher proportion of men undergoing the procedure than women and these studies have been conducted in various countries (Hariz et al., 2003, 2013; Mathkour et al., 2017; Deshpande et al., 2022). These findings emphasize the need for additional study regarding potential unconscious bias among healthcare providers, variations in the clinical indications for surgery based on gender, concerns specific to women regarding surgical complications, and gender-specific coping mechanisms for managing disease symptoms.

Referral bias due to gender has been identified as a contributing factor to the observed gender disparities in the utilization of DBS for individuals with PD (Jost et al., 2022). Specifically, women with PD are less likely to be referred for DBS evaluation by general practitioners and neurologists, leading to fewer women undergoing DBS surgery. This raises the question of whether the lack of diversity in the medical field plays a role in these disparities. Increasing the representation of women in movement disorders and neurosurgery specialties is one potential approach to address this disparity. Additionally, research on patient preferences for physician characteristics has shown that female patients prefer gender-concordant providers, highlighting the significance of promoting diverse representation within healthcare settings (García et al., 2003).

Differences in the clinical presentation and progression of PD have been observed between men and women, with women tending to experience longer disease duration, greater severity of dyskinesia, and more reduction in motor scores with medication (Haaxma et al., 2007; Shpiner et al., 2019). These variations in clinical phenotype may influence the decision to undergo DBS and contribute to the observed gender disparities. However, when it comes to clinical outcomes and responses to DBS, existing research suggests that men and women show comparable results in terms of quality of life, motor symptoms, medication needs, and motor outcomes (Chandran et al., 2014; Shpiner et al., 2019; Deshpande et al., 2022; Jost et al., 2022). These findings suggest that nonclinical factors likely play a role in the observed gender gap in DBS utilization.

Nonclinical factors, such as patient self-selection and preferences, have been found to contribute to the gender disparities in DBS utilization. Female patients often cite personal preferences and a heightened fear of complications as reasons for not pursuing DBS (Hamberg and Hariz, 2014; Shpiner et al., 2019), despite the same complication rates between men and women. One possible explanation for this is related to risk-taking behavior, as studies in psychology have shown that men exhibit a higher propensity for risk-taking behavior (Rolison et al., 2014). Additionally, individual factors and follow-up patterns further contribute to the complex interplay between gender and the decision-making processes related to DBS utilization. For example, the caregiving dynamics for women with PD differ, as they are less likely to rely on a spouse as their primary caregiver, more inclined to employ paid caregivers, and frequently attend appointments independently (Dahodwala et al., 2018). Incorporating social work consults into their care plan and ensuring access to home healthcare services could offer valuable support in addressing these distinctive needs and challenges.

To address the gender disparities in DBS utilization, promoting awareness and education about DBS among women with PD is crucial. Providing accurate information can help address misconceptions or concerns and empower women to make informed decisions about their treatment options. The insights gained from the Parkinson Foundation Women and PD TALK PCORI project (Parkinson's Foundation, 2019) can be utilized to tackle DBS disparities by tailoring care approaches to address women’s specific priorities, leading to improved treatment outcomes. Furthermore, this valuable information can guide researchers in studying gender-related factors, bridging knowledge gaps, and promoting equitable access to resources and support for women with PD. Future research should focus on exploring the decision-making processes of women with PD and investigating the clinical reasoning behind the referral patterns of general practitioners and neurologists. By understanding these factors more deeply, interventions can be developed to address and potentially mitigate the gender disparities in DBS utilization (Jost et al., 2022).

### Socioeconomic disparities

Socioeconomic status encompasses factors such as income, access to transportation, social support, and the ability to take time off work for surgery and follow-up visits. Several studies conducted in various countries have identified socioeconomic status as a significant determinant of DBS utilization in patients with PD (Chandran et al., 2014; Henriksen et al., 2020; Cramer et al., 2022). These studies show that higher household incomes are associated with a greater likelihood of receiving DBS and achieving better functional outcomes compared to patients with lower socioeconomic status (Willis et al., 2014; Cramer et al., 2022). This may bias referrals toward patients with more significant financial resources, potentially leading to disparities in access to DBS and subsequent functional outcomes (Cramer et al., 2022).

In developed countries, Medicaid coverage, which is often associated with lower socioeconomic status, has been identified as a potential reason for lower rates of DBS surgeries among Black patients with PD (Chan et al., 2014). Conversely, in low-income and lower-middle-income countries, financial constraints, lack of insurance coverage, out-of-pocket expenditures, limited referrals, inadequate access to infrastructure, and the absence of multidisciplinary DBS teams have been identified as barriers to DBS utilization (Chandran et al., 2014; Jourdain and Schechtmann, 2014).

One potential solution to address the socioeconomic disparity in DBS utilization is to make DBS systems more affordable, particularly in developing countries. The high costs associated with DBS devices and procedures create barriers to access for individuals with limited financial resources. Lowering the prices of DBS systems would enhance accessibility for individuals from lower socioeconomic backgrounds (Zhang et al., 2020). For policymakers, government initiatives similar to Imran Khan’s health insurance plan (“Sehat Sahulat program”) that aims to provide health insurance coverage to low-income households can provide access to medical services at partner hospitals and healthcare facilities without incurring out-of-pocket expenses. This alleviates the financial burden on poor individuals seeking medical treatment in Pakistan could be implemented to improve access to DBS for individuals from lower socioeconomic backgrounds (Barber and Shahza, 2022).

Furthermore, alternative technologies such as high-intensity focused ultrasound (HiFU) may offer a potential solution (Krishna et al., 2023), as the expenses and post-surgical care associated with HiFU are less than DBS. Additionally, exploring the feasibility of performing HiFU by movement disorders-trained neurologists and neuroradiologists could expand access to this latest technology to provide surgical therapies for individuals in developing countries without neurosurgeons.

Surprisingly, despite having free public healthcare systems in developed countries like Denmark and Canada, disparities in the availability of DBS for PD still exist (Crispo et al., 2020; Henriksen et al., 2020). Regional variations in the distribution of neurologists and neurosurgeons are considered significant barriers to accessing DBS treatment. Referral patterns and disparities in the availability of movement disorders specialists and neurosurgeons across different regions are potential factors contributing to the limited availability of DBS for PD patients. Thus, it is crucial to take measures to increase the number of neurosurgeons and movement disorders-trained neurologists, especially in regions where specialist care is less accessible, in order to mitigate this disparity.

### Limitations

This systematic review is based on studies with limitations that must be acknowledged. Most studies are derived from single-center experiences (Chandran et al., 2014; Shpiner et al., 2019; Deshpande et al., 2022; Watanabe et al., 2022; Skelton et al., 2023), capturing only the final stages of the extensive clinical pathway for DBS surgeries and may restrict the generalizability of the findings. The small sample sizes in some studies limit the statistical power of the primary outcomes (Shpiner et al., 2019; Deshpande et al., 2022; Watanabe et al., 2022; Skelton et al., 2023). Additionally, using the NIS dataset (Chan et al., 2014; Willis et al., 2014; Cramer et al., 2022; Dorritie et al., 2023), which is retrospective, introduces limitations related to coding accuracy and data completeness. Furthermore, the cross-sectional study designs employed in several studies hinder the systematic assessment of the reasons behind DBS disparities. Moreover, it is important to note that highly selected patients were included in certain studies for good surgical outcomes, which may not be ideal for assessing gender differences in resource-poor countries (Chandran et al., 2014). Furthermore, surveys sent to societies to collect data on low and middle-income countries may have missed surgeons who are not society members or do not publish articles, potentially impacting the representation of the sample (Jourdain and Schechtmann, 2014).

Future studies should address these limitations by incorporating longitudinal designs to assess long-term outcomes, including the durability of benefits, disparities in follow-up care, and patient-reported outcomes such as quality of life and functional outcomes. Additionally, qualitative research should be conducted to gain insights into the experiences, perspectives, and decision-making processes of individuals from diverse populations, uncovering contextual factors, cultural beliefs, and social determinants that contribute to disparities and informing tailored interventions.

## Conclusion

In summary, this study identified a range of factors that contribute to disparities in the utilization of DBS for PD, encompassing racial, gender, and socioeconomic disparities, as well as considerations related to financial constraints, geographic factors, education level, and healthcare-seeking behavior. Future research must acknowledge and address the limitations of existing studies, explore the intersectionality of these factors, and develop potential strategies to enhance equity in DBS therapy for individuals with PD. Efforts should be directed toward improving physician behavior, mitigating marketing disparities, promoting cultural sensitivity in healthcare delivery, and investigating the interplay between socioeconomic factors and healthcare utilization. By adopting a comprehensive approach, healthcare systems can strive to eliminate disparities and enhance the overall surgical management of PD.

## Data availability statement

The original contributions presented in the study are included in the article/Supplementary Material, further inquiries can be directed to the corresponding author.

## Author contributions

AM: Conceptualization, Data curation, Formal analysis, Investigation, Methodology, Project administration, Writing – original draft, Writing – review & editing. KG: Data curation, Formal analysis, Methodology, Writing – review & editing. JM: Data curation, Formal analysis, Methodology, Writing – review & editing. RB: Methodology, Writing – original draft, Supervision. MF: Supervision, Writing – review & editing. CC: Supervision, Visualization, Writing – review & editing. SM: Supervision, Writing – review & editing. AA: Conceptualization, Formal analysis, Methodology, Supervision, Writing – review & editing.
