# Supplementary material for: A systematic review of health disparities research in deep brain stimulation surgery for Parkinson’s disease
Source: Front Hum Neurosci. 2023 Oct 27;17:1269401. doi: 10.3389/fnhum.2023.1269401 (PMC10641459; doi:10.3389/fnhum.2023.1269401)
Supplement: Supplementary file 1 [file Data_Sheet_1.docx]

Electronic Supplementary Material Appendix S1

Bibliographic Literature searching

| **Database** | **Hits** |
| --- | --- |
| Embase | 664 |
| PubMed | 762 |
| Cochrane Library | 458 |
| Web of Science | 305 |
| Total | 2189 |
| - duplicates | 510 |
| Unique studies to screen | 1679 |

Database: Embase

Host: Elsevier

Data Parameters: 1947 to Present

Date Searched: September 12, 2023

Searcher: Rebecca Billings                        Strategy Checked By: Emma O'Hagan

Search Strategy:

| **#** | **Searches** | **Results** |
| --- | --- | --- |
| 1 | ('health disparity'/de OR 'vulnerable population'/exp OR 'social determinants of health'/de OR (disparit* OR discriminat* OR underrepresent* OR underserved OR marginalized OR inclusiv* OR inclusion  OR ((age OR cultural* OR economic* OR emigrant* OR emigrat* OR ethnic* OR ethno* OR financ* OR gender* OR global* OR health OR immigrant* OR immigrat* OR income OR insurab* OR insurance OR insure* OR minorit* OR multiethnic* OR race OR racial* OR sex OR social* OR socio-economic OR sociodemographic* OR socioeconomic OR status OR vulnerable) NEAR/4 (barrier* OR disparat* OR disadvantage* OR inequality* OR inequit* OR equit* OR discriminat* OR differen*)) OR ((vulnerable OR disadvantage*) NEAR/4 (person* OR people* OR population* OR minorit* OR group*)) OR ((social) NEAR/3 (determin*))):ti,ab,kw) | 1689688 |
| 2 | ('deep brain stimulator'/exp OR 'brain depth stimulation'/de OR 'deep brain stimulation electrode'/exp OR (DBS OR cerebellar OR subthalamic OR ((deep-brain OR brain-depth) NEAR/4 (surger* OR stimulat* OR excitation OR stimul* OR procedur* OR program* OR treat* OR therap* OR implant* OR activa OR kinetra OR medtronic OR soletra OR suretek OR vercise OR percept* OR electrode))):ab,ti,kw) | 165768 |
| 3 | ('parkinson disease'/exp OR (PD OR Parkinson* OR hemiparkinsonism OR ((movement-disorder* OR paralysis-antigens) NEAR/4 (disease* OR lewy OR idiopathic OR syndrome* OR disorder*))):ab,ti,kw) | 491938 |
| 4 | #1 AND #2 AND #3 | 1294 |
| 5 | [english]/lim NOT ([conference abstract]/lim OR [conference paper]/lim OR [conference review]/lim) | 31805842 |
| 6 | #4 AND #5 | 664 |

Notes: Copy/paste search into the advanced query in Embase.com.

Dataset saved as: Memon_DeepBrainParkinsons664.ris

Database: PubMed

Host: National Library of Medicine (<https://www.nlm.nih.gov/>)

Data Parameters: 1946 to Present

Date Searched: September 12, 2023

Searcher: Rebecca Billings                      

Search Strategy:

| **#** | **Searches** | **Results** |
| --- | --- | --- |
| 1 | (("Healthcare Disparities"[Mesh] OR "Health Inequities"[Mesh] OR "Minority Health"[Mesh] OR "Health Disparate, Minority and Vulnerable Populations"[Mesh] OR disparit*[tiab] OR barriers[tiab] OR disparat*[tiab] OR disadvantage*[tiab] OR inequality*[tiab] OR inequit*[tiab] OR equit*[tiab] OR inclusiv*[tiab] OR inclusion[tiab] OR discriminat*[tiab] OR underrepresent*[tiab] OR underserved[tiab] OR marginalized[tiab] OR sociodemographic*[tiab] OR minorit*[tiab] OR ethnic*[tiab] OR multiethnic*[tiab] OR ethno*[tiab] OR immigrant*[tiab] OR emigrant*[tiab] OR immigrat*[tiab] OR emigrat*[tiab] OR vulnerable[tiab] OR gender*[tiab] OR sex[tiab] OR race[tiab] OR racial*[tiab] OR age[ti])) | 2766995 |
| 2 | ("Deep Brain Stimulation"[Mesh] OR "Electric Stimulation Therapy"[Majr:NoExp] OR ((deep-brain-stimulat*[tiab] OR brain-depth[tiab] OR DBS[tiab] OR cerebellar[tiab] OR subthalamic[tiab]) AND (surger*[tiab] OR stimulat*[tiab] OR excitation[tiab] OR electrotherap*[tiab] OR electrical[tiab] OR stimulus[tiab] OR procedur*[tiab] OR treatment*[tiab] OR therap*[tiab] OR implant*[tiab] OR kinetra[tiab] OR Medtronic[tiab] OR soletra[tiab] OR suretek[tiab] OR vercise[tiab]))) | 60613 |
| 3 | ("Parkinsonian Disorders"[Mesh] OR PD[tiab] OR Parkinson*[tiab] OR hemiparkinsonism[tiab] OR movement-disorder*[tiab] OR paralysis-agitans[tiab] OR lewy-body[tiab] OR Ramsay-Hunt[tiab]) | 306104 |
| 4 | #1 AND #2 AND #3 | 762 |

Notes: N/A

Dataset saved as: Memon_DeepBrainParkinsons762.nbib

Database: Cochrane Library

Host: Wiley

Data Parameters: 1998 to Present

Date Searched: September 12, 2023

Searcher: Rebecca Billings                    

Search Strategy:

| **#** | **Searches** | **Results** |
| --- | --- | --- |
| 1 | MeSH descriptor: [Healthcare Disparities] this term only | 277 |
| 2 | MeSH descriptor: [Health Inequities] explode all trees | 256 |
| 3 | MeSH descriptor: [Health Disparate, Minority and Vulnerable Populations] explode all trees | 7116 |
| 4 | MeSH descriptor: [Social Determinants of Health] this term only | 79 |
| 5 | (disparit* OR disparat* OR disadvantage* OR inequality* OR inequit* OR equit* OR barrier* OR discriminat* OR underrepresent* OR underserved OR marginalized OR socioeconomic* OR sociodemographic* OR minorit* OR ethnic* OR multiethnic* OR immigrant* OR emigrant* OR vulnerable OR gender* OR race OR racial*):ti | 12438 |
| 6 | #1 OR #2 OR #3 OR $4 OR #5 | 962011 |
| 7 | MeSH descriptor: [Deep Brain Stimulation] this term only | 430 |
| 8 | (DBS OR deep-brain OR brain-depth) AND (surger* OR stimulation) | 2850 |
| 9 | #7 OR #8 | 2850 |
| 10 | MeSH descriptor: [Parkinson Disease] explode all trees | 6215 |
| 11 | ((PD OR Parkinson* OR hemiparkinsonism)):ti,ab,kw | 48797 |
| 12 | #10 OR #11 | 48797 |
| 13 | #6 AND #9 AND #12 | 458 |

Notes: Built search using Advanced Search->Search Manager to enter subject headings and keyword terms and combining the concepts using line-by-line formatting.

Dataset saved as: Memon_Cochrane458.ris

Database: Web of Science

Host: Clarivate

Data Parameters: 1990 to Present

Date Searched: September 12, 2023

Searcher: Rebecca Billings

Search Strategy:

| **#** | **Searches** | **Results** |
| --- | --- | --- |
| 1 | (disparit* OR barriers OR disparat* OR inequality* OR inequit* OR equit* OR inclusiv* OR discriminat* OR underrepresent* OR underserved OR marginalized OR minorit* OR ethnic* OR multiethnic* OR immigrant* OR emigrant* OR vulnerable-population* OR racial*) | 2185186 |
| 2 | (deep-brain OR brain-depth OR DBS OR cerebellar OR subthalamic) AND (surger* OR stimulat* OR excitation OR device* OR electrotherap* OR electrical OR stimulus OR implant*) | 41490 |
| 3 | (PD OR Parkinson* OR hemiparkinsonism OR lewy-body OR Ramsay-Hunt) | 478168 |
| 4 | #1 AND #2 AND #3 | 414 |
| 5 | Filters applied: Article | 305 |

Notes: Searched in Web of Science Core Collection, editions included: Science Citation Index Expanded (SCI-EXPANDED) 1990-present; Social Sciences Citation Index (SSCI) 1990-present; & Emerging Sources Citation Index (ESCI) 2018-present.

Under the Documents Search, each search concept was searched as a Topic search separately, then combined using the Advanced search history.

Dataset saved as: Memon_WOS305.ris; Memon_PubMed762.xml
